# ASO Author Reflections: Is the VATS Technique Superior to Thoracotomy Techniques in Terms of Respiratory Muscle Function and Functional Exercise Capacity?

**DOI:** 10.1245/s10434-024-15429-z

**Published:** 2024-05-16

**Authors:** Funda Sirakaya, Ebru Calik Kutukcu, Mehmet Ruhi Onur, Erkan Dikmen, Ulas Kumbasar, Serkan Uysal, Riza Dogan

**Affiliations:** 1 Faculty of Medicine, Department of Thoracic Surgery, Hacettepe University, Ankara, Turkey; 2 Faculty of Physical Therapy and Rehabilitation, Department of Cardiorespiratory Physiotherapy and Rehabilitation, Hacettepe University, Ankara, Turkey; 3 Faculty of Medicine, Department of Radiology, Hacettepe University, Ankara, Turkey

## Past

The most common surgery for non-small cell lung cancer is lobectomy, which can be performed through either thoracotomy or video-assisted thoracic surgery (VATS). The research examining changes in respiratory muscle function and exercise capacity after lobectomy performed with conventional thoracotomy (CT), muscle-sparing thoracotomy (MST), or VATS is insufficient. Lobectomy has negative effects on respiratory muscle function and exercise capacity, but the effects of surgical techniques on these parameters are not clear.^1–4^ Nomori et al.^2^ found no difference in exercise capacity or respiratory muscle strength between limited thoracotomy and VATS procedures for patients with lung cancer who undergo lobectomy. In another study, the CT group observed a greater decrease in respiratory muscle strength than the MST group after pulmonary resection.^3^ Bernard^4^ found that VATS results in better recovery of respiratory muscle function after lung biopsy than CT. The precise effects and origins of surgery-induced effects on respiratory muscle strength remain unclear. However, no study has examined the effects of all three surgical approaches to lobectomy on the respiratory muscle function and exercise capacity of patients with lung cancer.

## Present

Lobectomy negatively affects respiratory muscle strength, diaphragm thickness, and functional exercise capacity. The impact of VATS and MST surgical techniques on these parameters is similar, but VATS is better than CT. The MST group showed less impact on exercise capacity than the CT group. Additionally, the VATS group demonstrated lower depression and anxiety levels than the thoracotomy group. A statistically significant difference was detected only in the thickness of the diaphragm after inspiration on the side treated with surgery. However, all the groups experienced negative effects on respiratory muscle strength, functional exercise capacity, and diaphragm thickness compared with the preoperative period.

## Future

Thoracic surgeons should refer patients to physiotherapists before lobectomy, especially patients undergoing CT, for preoperative preparation and patient education, as well as to minimize the decrease in respiratory muscle functions and exercise capacity. Studies are needed to investigate the effectiveness of pre- and postoperative rehabilitation approaches for VATS, CT, and MST patients. If lobectomy with VATS would be technically difficult for a particular patient, MST may be an option preferable to CT because of its impact on exercise capacity.
